# Ana Antić, *Therapeutic Fascism: Experiencing the Violence of the Nazi New Order in Yugoslavia*

**DOI:** 10.1093/shm/hky009

**Published:** 2018-02-12

**Authors:** Sarah Marks

**Affiliations:** Birkbeck, University of London

In 1941, Yugoslavia was dismembered by the Axis powers. The collaborating governments and the occupiers began a violent campaign against political dissent, and the extermination of ethnic and social groups considered enemies of the fascist cause. Civilians—particularly those allied with the Communists—became the target of brutal attacks. Communist partisans themselves, led by Josip Tito, fought against the occupation in one of the most ideologically polarised conflicts of the Second World War. Tito’s People’s Liberation Army captured Belgrade by November 1945, with the establishment of the socialist Federal People’s Republic of Yugoslavia. How was this extraordinarily violent conflict experienced by civilians, soldiers and partisans? And what role did the psychiatric profession play?

Antić’s book tells of the violence and trauma of the occupation at the level of the individual psyche, through 949 psychiatric case histories across varied hospital archives. The files unveil not only the psychological impact of wartime occupation, but also people’s internal conflicts about the political events surrounding them. They offer a unique insight into class, gender and political allegiances, making a striking, original contribution to the social history of Yugoslavia. The book also brings to light extraordinary histories of the overt political use of ‘therapeutic’ techniques. The pro-German Institute for compulsory re-education of youth in Smerderevska Palanka employed reformatory—and in some cases psychoanalytic—approaches to ‘cure’ students of their Communist proclivities. Conversely, after 1945 the Military Psycho-hygienic Institute in Kovin outside Belgrade developed a psychodynamic treatment programme for traumatised, demobilised soldiers suffering from ‘Partisan hysteria’, adapting to life as productive members of the Party.

Antić cites these examples as part of an over-arching theoretical shift in Yugoslav psychiatry: from a biological and hereditarian aetiological model to an understanding that incorporated the possibility of the reform of a personality, the role of the environment and a renewed therapeutic optimism—sometimes framed in psychoanalytic terms. The experience of the violence of the occupation was the key turning point in the transformation of the professional worldview. By reframing mental illness and treatment in socio-economic and environmental terms, postwar Yugoslav psychiatry found its new position resonated with that of the new socialist society. Whilst a detailed analysis of the socialist period goes beyond the scope of the monograph, one wonders to what degree the Yugoslav psychiatrists of the 1940s had access to international debates in psychiatry. A shift towards social psychiatry was simultaneously occurring elsewhere, as historians including Gerald Grob and Michael Staub have shown. Their comparative national studies uncovered the similarly transformative impact of war trauma on psychiatric professions elsewhere, particularly in the USA.

Antić’s meditation on the psychiatric case file as a historical source is, in and of itself, crucial reading for historians of the psy-disciplines. Drawing on the work of a diverse range of scholars such as James Glass, Elizabeth Lunbeck and Jonathan Sadowsky, the author also makes a strong case for the value of these sources for a much broader audience of cultural historians. Instead of dismissing the experience of the psychiatric patient, the content of psychotic and neurotic symptoms themselves offer an insight into political realm. Their unusual structure as mediated documents, based on interviews carried out by clinicians, necessitates attention to interpretive strategies from narrative theory. Citing Fanon, the author argues that these patient-orientated sources remind us of the inevitable psychiatric consequences of violence and colonialism. Psychiatry, Antić asserts, offers an alternative history of the brutality of fascism, but one that is no less central to understanding the experience of violent regimes than any other form of testimony.

There is a notable absence from this methodological discussion, however: it lacks an exploration of the ethical implications of the historical use of patient files. This is by no means limited to this book—it is a debate which historians of psychiatry as a profession seem to have somehow eschewed. Unlike Elizabeth Lunbeck’s analysis of Boston Asylum records in the nineteenth century, the documents available to Antić’s study are within living memory.^1^ While there is not a great deal of detailed personal information, it is plausible that enough information is provided to potentially make the patients identifiable. Many countries, Britain included, maintain a 100-year restriction on access to patient archives. But many others do not, and accessing medical files can be entirely legal in such contexts. It is worth reflecting on the implications of using these records for historical research, when consent was not granted by the patients themselves. This book, in many ways, serves as a robust vindication of the importance of bringing these patient stories to light; restoring an aspect of experience to the historical record, from actors who would not otherwise be given a voice. Indeed, Antić eloquently shows us that it is imperative to do so, to fully document and acknowledge the effects of wartime violence upon the psyche.

## Footnotes

1 Elizabeth Lunbeck, *The Psychiatric Persuasion; Knowledge, Gender, and Power in Modern America* (Princeton: Princeton University Press, 1994).

